# Detectability constraints on meso-scale structure in complex networks

**DOI:** 10.1371/journal.pone.0317670

**Published:** 2025-01-22

**Authors:** Rudy Arthur

**Affiliations:** Department of Computer Science, University of Exeter, Exeter, United Kingdom; University of Glasgow, UNITED KINGDOM OF GREAT BRITAIN AND NORTHERN IRELAND

## Abstract

Community, core-periphery, disassortative and other node partitions allow us to understand the organisation and function of large networks. In this work we study common meso-scale structures using the idea of block modularity. We find that the configuration model imposes strong restrictions on core-periphery and related structures in directed and undirected networks. We derive inequalities expressing when such structures can be detected under the configuration model which are closely related to the resolution limit. Nestedness is closely related to core-periphery and is similarly restricted to only be detectable under certain conditions. We then derive a general equivalence between optimising block modularity and maximum likelihood estimation of the parameters of the degree corrected Stochastic Block Model. This allows us to contrast the two approaches, how they formalise the structure detection problem and understand these constraints in inferential versus descriptive approaches to meso-scale structure detection.

## 1 Introduction

A common task in network science is the identification of intermediate or meso-scale structure by partitioning the nodes of the network into non-overlapping sets [1, 2]. The primary structure of interest has generally been assortative communities [3–6]. These are tightly connected groups of nodes which split the network into a number of weakly connected modules. Another meso-scale structures of interest is core-periphery [7–12], consisting of a core set and a periphery set, where the core is densely connected internally and to the periphery, while periphery nodes are predominantly connected to core nodes. There are also disassortative structures which have received somewhat less attention [13–16], but represent the other side of community structure, dense connections between groups and sparse connections within them.

Recent work [17–19] has studied hard-partitions of networks, where the non-overlapping groups are arranged in different meso-scale patterns. While this excludes structures like k-cores [20], rich-clubs [21] and overlapping communities [22] a hard partition is often a very useful way to get a bird’s eye view of the network. For a hard partition the network’s adjacency matrix has an approximate block structure. For small numbers of subsets (2 in [18] and 2,3,4 in [17]) it is feasible to enumerate all the possible block patterns: e.g. 3-community, tripartite, core-double periphery etc. In [18, 19] meso-scale structure is identified based on the ratio of existing to possible links within and between communities. [17] bases meso-scale structure detection on the excess or deficit of links within or between blocks compared to a null model, we will follow the latter approach in this work.

While enumerating block patterns [17] also found that certain intuitively expected ones, notably the 2 × 2 core-periphery structure, are ‘undetectable’. To briefly summarise the key finding of [17], consider a network split into two parts, a core and periphery, labelled *c* and *p*, with the number of edges in and between parts *S*_*cc*_, *S*_*cp*_ = *S*_*pc*_, *S*_*pp*_ (precise definitions will be given in Section 2). In the configuration model, each node has the same degree as the original so, comparing the observed network to its configuration model, we have the identities

$$\begin{array}{c} S_{cc}+S_{cp}=E\left[S_{cc}^{conf}\right]+E\left[S_{cp}^{conf}\right] \end{array}$$ (1)

$$\begin{array}{c} S_{pc}+S_{pp}=E\left[S_{pc}^{conf}\right]+E\left[S_{pp}^{conf}\right] \end{array}$$ (2)

Where $E[S_{cp}^{conf}]$ etc. are the expected number of edges in and between blocks in any network labelled in the same way, with the same degree sequence as the original. Rearranging gives

$$\begin{array}{c} S_{cc}-E\left[S_{cc}^{conf}\right]=S_{pp}-E\left[S_{pp}^{conf}\right] \end{array}$$ (3)

This implies that it is impossible for a network to be built from two blocks where the core block has more edges than the configuration model (left hand side positive) and, simultaneously, the periphery block has fewer edges (right hand side negative). The first aim of this work is to extend this result, showing under what conditions different meso-scale patterns are undetectable i.e. when the configuration model precludes structures or only allows them in certain regimes.

To do this I will use a generalised formulation of modularity called *block modularity* [23]. This extends Newman’s original modularity definition [24, 25] in a way that allows the evaluation of partition quality for a variety of meso-scale structures. Block modularity captures the, intuitively reasonable, notion that for a meso-scale structure to be present requires that there be more links within or between the corresponding blocks than in a random network, similarly to Eq 3. A key point is that the ‘random network’ is one selected from a particular null model. While numerous choices for a null model are possible [26, 27], generally the configuration model [28] is used.

A random network drawn from the configuration model is one with the same degree sequence as the observed network. Degree distribution along with correlations and anti-correlations among node degrees have long been recognised as key characteristics that explain many of the properties of complex networks [1, 29–31]. An excess or deficit of edges with respect to the configuration model implies that there is structure in the network which cannot be explained by the node degrees alone. This requirement of structure beyond that implied by the degree sequence turns out to be quite rigid, and imposes hard constraints on the types of meso-scale structures which are possible.

This work does not develop an algorithm to find ‘good labels’ or evaluate other algorithms that do so, see [32, 33] for this. Rather, we aim systematise and generalise results like Eq 3. Such results tell us that structures which would seem to be intuitive are also present in a randomly rewired network. The consequence of this is not that some community detection methods don’t give us the labels we expect, but rather that certain structures may not actually be meaningful under strong null hypotheses, somewhat analogously to the fact that significant correlation can become non-significant after accounting for spatial or temporal autocorrelation.

The reader familiar with the community detection literature will be aware that inferential methods based on the Stochastic Block Model (SBM) solve many of the issues with modularity based community detection [34]. The question may arise as to why one should consider modularity, even a generalised form of it, given effective alternatives. Firstly, I note that modularity optimisation is still an extremely popular method of community detection in applications and still an active research topic [35–38]. Furthermore, it is only recently that the community detection literature has begun to address the importance and ubiquity of non-assortative partitions [19]. Methods for detecting these structures are often based on modularity-like functions [11, 16, 39–41] so, a deeper understanding of such functions can be useful to improve these methods. However, issues with modularity remain and the second aim of this work is to understand the connection of modularity maximisation to SBM inference, in particular how constraints on meso-scale structure, like Eq 3, map to that framework.

Constraints like Eq 3 and others derived below have implications beyond the performance of some particular optimisation algorithm and force us to examine deeper questions. In particular, there is a tension between the SBM, where there are no such constraints, and modularity where there are. This is examined in Section 6, where a connection between this paper’s methods and SBM model inference is described. We show that for a core-periphery network SBM inference can indeed find the ‘correct’ node labels while *at the same time* the majority of networks with the observed degree distribution are also core-periphery. This is a similar sort of observation as [42] who find that node degrees alone can explain patterns in clustering coefficients. This paper aims to demonstrate similar results for meso-scale structure as well as to understand how different formulations of the community/structure detection problem can lead to different conclusions about the same networks.

The contributions of the paper are the following:

1. Generalised modularity is discussed and extended to directed networks.
2. 2 × 2 block patterns in undirected and directed networks are enumerated. We identify two directed patterns (Basin-Delta and Community Hierarchy) which do not seem to have been discussed in the literature before.
3. Inequalities are derived showing which meso-scale structures are detectable under the configuration model.
4. We complete the connection of [43] between modularity maximisation and SBM likelihood maximisation, going beyond the planted partition model to show how any SBM can be mapped to a modularity maximisation problem and *vice versa*.
5. We study a specific case of CP structure, contrasting SBM model inference with modularity maximisation.

The layout of the paper is as follows, in Section 2 we reintroduce block modularity and extend the definition to directed networks. In order to constrain structures, we have to know which ones are possible, this is examined in Section 3 where all possible 2 × 2 block patterns for directed and undirected networks are enumerated. We then use block modularity to derive the constraints found by [17] in a somewhat simpler way, as well as extending those results to the directed case. In Section 4 we derive further restrictions on smaller meso-scale patterns as part of larger networks. These constraints generalise the well known resolution limit [44]. Section 5 discusses the possibility of ‘nested’ networks [45] under the configuration model. Section 6 considers the degree corrected Stochastic Block Model (dc-SBM) [46] and shows how this is related to Block Modularity. We also look at detecting CP structure from the modularity and SBM perspectives and discuss this further in Section 7.

## 2 Block modularity

Modularity was introduced by Newman [24] to measure the quality of a partition of a network into communities.

$$\begin{array}{c} Q_{Newman}=\frac{1}{2E}\sum_{ij}(A_{ij}-P_{ij})\delta \left(c\left(i\right),c\left(j\right)\right) \end{array}$$ (4)

The sums are over all *N* nodes of the network, *A* is the adjacency matrix, 2*E* = ∑_*ij*_ *A*_*ij*_ is twice the number of edges, *c*(*i*) is a function which returns the community label of the node *i* and *P*_*ij*_ is the expected number of links between *i* and *j* in the null model. With the configuration model as the null model

$$P_{ij}=\frac{k_{i}k_{j}}{2E}$$

where *k*_*i*_ = ∑_*j*_*A*_*ij*_ is the degree of *i*. An Erdős-Rènyi (ER) null model, which assumes a constant probability for every edge to exist (including self edges) has

$$P_{ij}=p=\frac{2E}{N^{2}}$$

Another common null model is the scaled configuration model [47]

$$P_{ij}=\gamma \frac{k_{i}k_{j}}{2E}$$

where *γ* is an arbitrary parameter. We will primarily deal with the configuration model and discuss the ER and scaled configuration model in S1 File. The stochastic block model (SBM) [48] is also often used to investigate meso-scale structure, though typically not as a null model, and we treat this separately in Section 6.

Modularity can be rewritten in terms of the adjacency matrix of the network induced by the partition. Define

$$S_{ab}=\sum_{i\in a,j\in b}A_{ij}$$

as the number of edges going between *a* and *b*, or twice the number of edges inside *a* if *a* = *b*. Similarly for the null model define,

$$S_{ab}^{P}=\sum_{i\in a,j\in b}P_{ij}.$$

Eq 4 becomes

$$\begin{array}{c} Q_{Newman}=\frac{1}{2E}\sum_{a}(S_{aa}-S_{aa}^{P}) \end{array}$$ (5)

where the sum is over all *K* sets in the partition. Written like this, *Q*_*Newman*_ is the sum over groups of the excess or deficit of edges in the observed network compared to the number predicted by the null model. For the configuration null

$$Q_{Newman}=\frac{1}{2E}\sum_{a}^{K}(S_{aa}-\frac{T_{a}^{2}}{2E})$$

with

$$T_{a}=\sum_{i\in a}k_{i}$$

[23] introduced a generalisation of this function (see also [11]), with

$$Q_{ab}=S_{ab}-S_{ab}^{P}$$

the *block modularity* is defined as

$$\begin{array}{c} Q\left(B\right)=\frac{1}{2E}\sum_{ab}^{K}Q_{ab}B_{ab} \end{array}$$ (6)

with the *block matrix* *B* having entries equal to ±1. For the configuration model,

$$S_{ab}^{P}=\frac{T_{a}T_{b}}{2E}.$$

The target meso-scale structure is encoded in the block matrix *B*. For example, if the matrix *B* has 1s on on the diagonal and -1s elsewhere *Q*(*B*) = 2*Q*_*Newman*_ and *Q*(*B*) evaluates how well a partition groups the nodes into assortative communities. We are however, not limited to assortative communities. For example, if

$$B=(\begin{array}{cccc} 1 & 1 & -1 & -1\\ 1 & -1 & -1 & -1\\ -1 & -1 & 1 & 1\\ -1 & -1 & 1 & -1 \end{array})$$

then *Q*(*B*) evaluates how unexpected a partition of the nodes into a pair of core-peripheries is compared to the null model. [23] explores simultaneously optimising both the partition *c*(*i*) and the block pattern *B*, so that the meso-scale structure is discovered from the data and in Section 6 we will discuss an algorithm for determining *B* if the entries are treated as free parameters. However, finding labels by optimising *Q*(*B*) is not the aim of this work and we can simply select the values of *B* and use *Q*(*B*) to probe the network for the structure it represents.

### 2.1 Directed networks

The above can be extended straightforwardly to directed networks in much the same way as *Q*_*Newman*_ is extended in [49]. Let *A* be the adjacency matrix of a directed graph. Recall, in the directed case *A*_*ij*_ ≠ *A*_*ji*_. With

$$\begin{array}{c} k_{j}^{\left(in\right)}=\sum_{i}A_{ij},\;k_{i}^{\left(out\right)}=\sum_{j}A_{ij},\;E=\sum_{ij}A_{ij} \end{array}$$

Define

$$\begin{array}{c} Q_{↔}\left(B\right)=\frac{1}{E}\sum_{ab}^{K}Q_{ab}B_{ab} \end{array}$$ (7)

with *Q*_*ab*_ and *B*_*ab*_ as before, noting that in the directed case these need not be symmetric. Using the directed configuration model as the null model

$$\begin{array}{c} S_{ab}^{P}=\frac{T_{a}^{\left(out\right)}T_{b}^{\left(in\right)}}{E},\;T_{a}^{\left(out\right)}=\sum_{i\in a}k_{i}^{\left(out\right)},\;T_{b}^{\left(in\right)}=\sum_{j\in b}k_{j}^{\left(in\right)} \end{array}$$

## 3 Allowed block patterns

**Fig 1:**
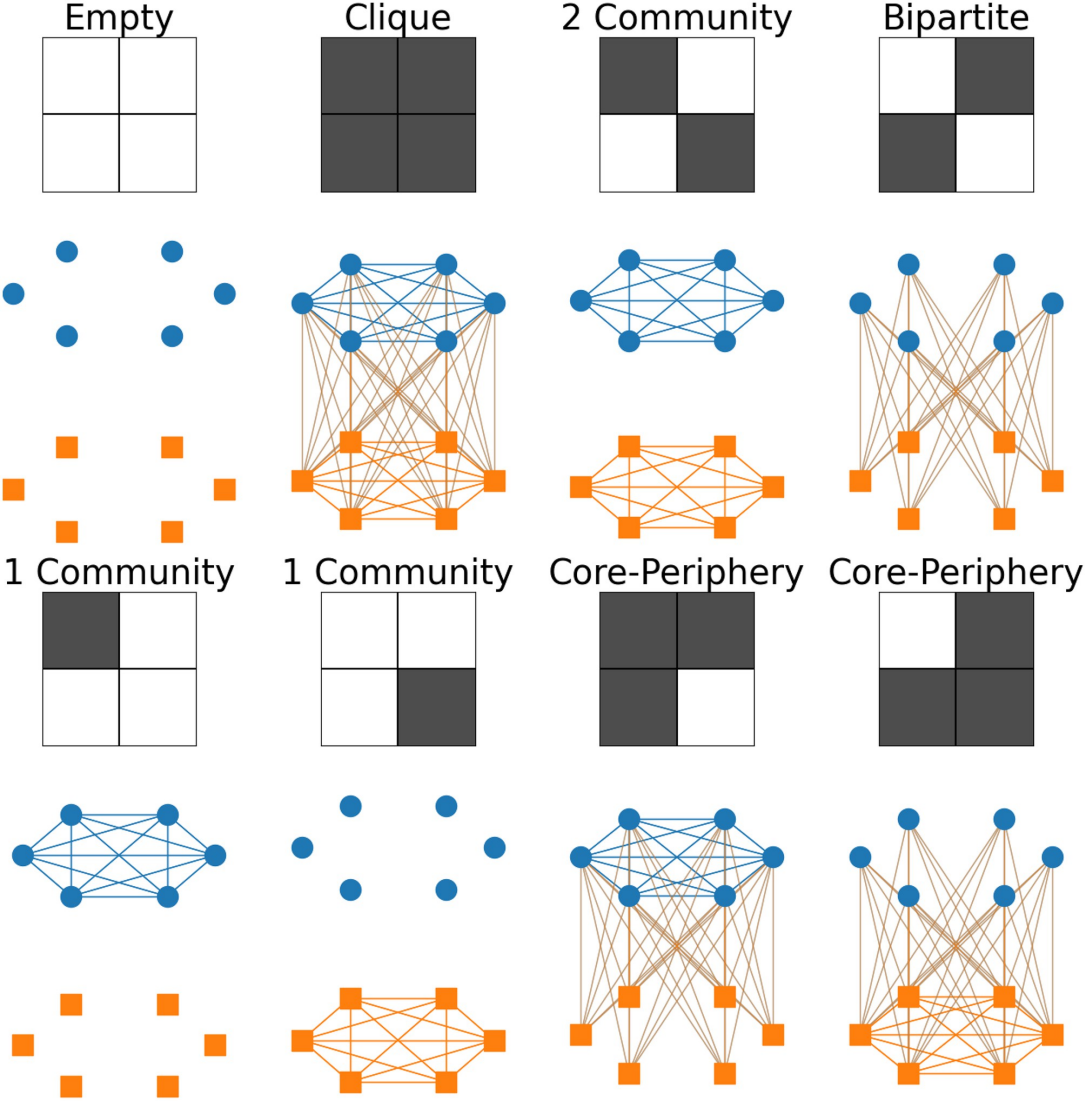
All 2x2 block patterns for undirected networks and an example network realising that block pattern. The pairs with the same name are equivalent but both are shown for completeness.

**Fig 2:**
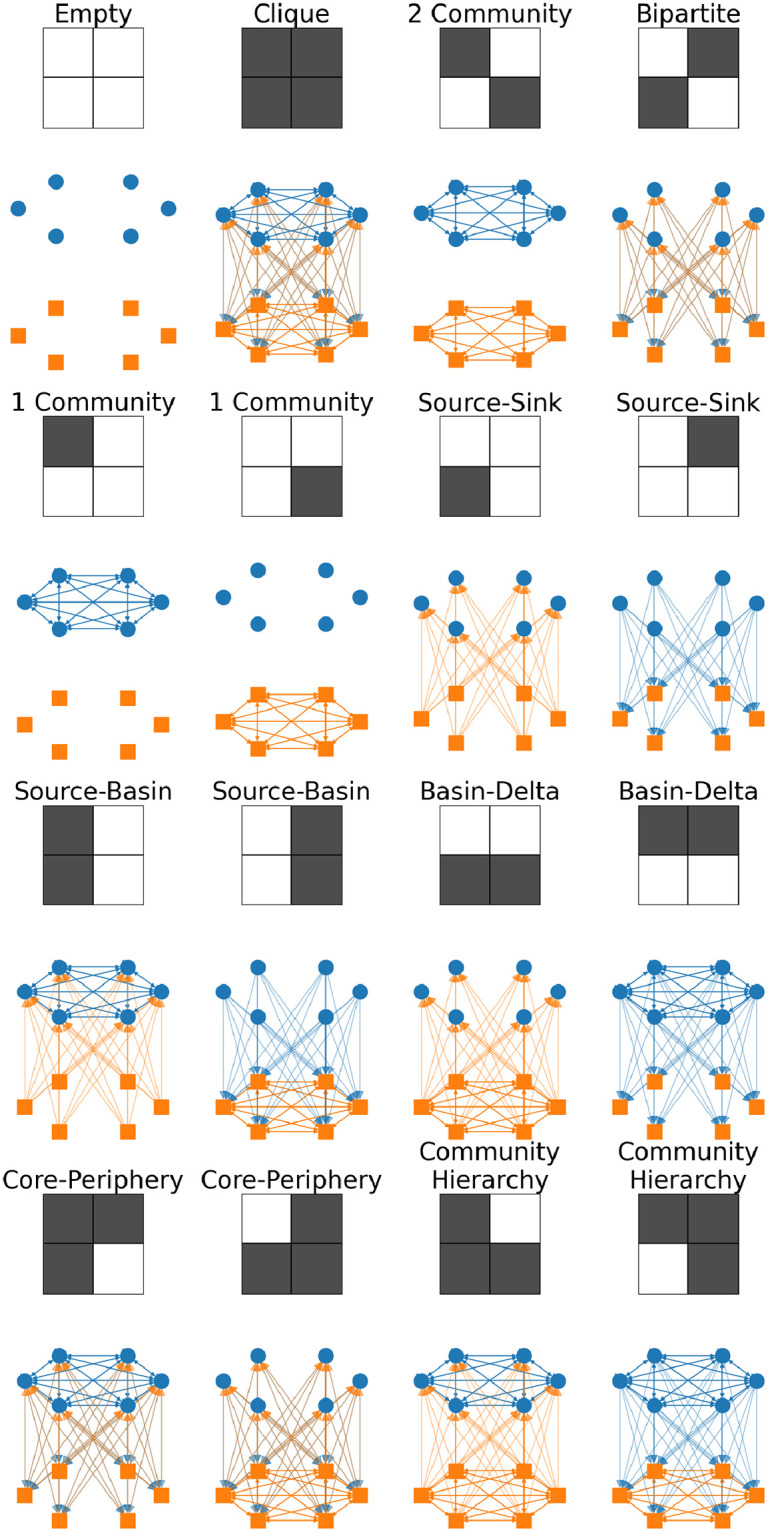
All 2x2 block patterns for directed networks and an example network realising that block pattern. The pairs with the same name are equivalent but both are shown for completeness.

All possible 2 × 2 block matrices *B* with entries ±1 are shown in Fig 1 for the undirected and Fig 2 for the directed case, where a black square is +1 and a white square −1, corresponding to an excess or deficit of edges respectively. The example networks show the limit where the + 1 blocks are fully connected and the −1 blocks have no edges. [19] groups the bottom 8 directed networks of Fig 2 into two classes, source-basin and core-periphery. Here we further divide those groups into

- **Source-Basin**: one-way flow from a loosely connected group to a densely connected group.
- **Basin-Delta**: one-way flow from a densely connected group to a loosely connected group.
- **Core-Periphery**: two-way flow between a densely connected group and a loosely connected group.
- **Community-Hierarchy**: one-way flow between densely connected groups.

### 3.1 Detectable 2 × 2 patterns

In the undirected case with the configuration model null

$$\begin{array}{cc} \sum_{b}Q_{ab} & =\sum_{b}S_{ab}-\frac{T_{a}}{2E}\sum_{b}T_{b}\\ & =\sum_{i\in a,j}A_{ij}-\frac{T_{a}}{2E}\sum_{i\in a,j}k_{j}\\ & =\sum_{i\in a}k_{i}-T_{a}=0 \end{array}$$ (8)

This implies the row and column sums of the *Q*_*ab*_ matrix can’t simultaneously all be positive or all negative, which is equivalent to the result found by [17], Eq 3. This means that we can’t have a solid black or solid white, row or column (see the matrices of Fig 1) otherwise *Q*(*B*) is identically zero. In other words, it is impossible to have an excess (or deficit) of connections between one group and all the other groups under the configuration model null. I will refer to this as the KM rule.

An almost exactly analogous calculation applies in the directed case

$$\begin{array}{cc} \sum_{b}Q_{ab} & =\sum_{b}S_{ab}-\frac{T_{a}^{\left(out\right)}}{E}\sum_{b}T_{b}^{\left(in\right)}\\ & =\sum_{i\in a,j}A_{ij}-\frac{T_{a}^{\left(out\right)}}{E}\sum_{i\in a,j}k_{j}^{\left(in\right)}\\ & =\sum_{i\in a}k_{i}^{\left(out\right)}-T_{a}^{\left(out\right)}=0 \end{array}$$

and similarly for the column sums. Thus in both the directed and undirected cases, under the configuration model the only detectable 2 × 2 block patterns are 2-Community and Bipartite (called disassortative in [19]) networks

$$\left(\begin{array}{cc} 1 & -1\\ -1 & 1 \end{array})\;(\begin{array}{cc} -1 & 1\\ 1 & -1 \end{array}\right)$$

In particular a 2 × 2 Core-Periphery is not detectable i.e. the pattern

$$\left(\begin{array}{cc} 1 & 1\\ 1 & -1 \end{array}\right)$$

of excesses and deficits can’t be realised for any labelling.

With more than two blocks, other patterns can be observed, provided that the KM rules are satisfied. [17] provides a list of all allowed 3 × 3 and 4 × 4 patterns for the undirected case. To see that there must be more than just these row and column sum constraints, consider a pattern like

$$\left(\begin{array}{ccc} 1 & 1 & -1\\ 1 & -1 & -1\\ -1 & -1 & 1 \end{array}\right)$$

which represents a CP+1 community structure. This satisfies all the KM rules, however these rules say nothing about the sizes of the communities. Thus, an arbitrarily large 2 × 2 CP structure plus one isolated node would be allowed! We investigate the resolution of this paradox in the next section.

## 4 Constraint equations

We will mostly consider the undirected case using the configuration model null, since this is the most common, and interesting, case. Other cases are considered in S1 File. In the modularity framework, in order to say a structure is really present, there should be an excess or deficit of connections within or between groups compared to a random network. The structure we are aiming to detect is encoded by *B*. So, for example, assuming groups *c* and *p* formed a CP pair (as part of a larger network in order to satisfy the KM rules) we would require for those groups

$$Q_{cc}>0,Q_{cp}>0,Q_{pp}<0$$

simultaneously. That is, an excess of connections within the core, from core to periphery and a deficit of connections within the periphery. We can use the generalised modularity to summarise these requirements. We say a network has structure *B* (for some labelling) if all of the terms *Q*_*ab*_*B*_*ab*_ > 0. Since *Q*(*B*) = ∑_*ab*_ *Q*_*ab*_*B*_*ab*_ this is equivalent to saying that each block or pair or blocks must make a positive contribution to modularity. If it is not possible to arrange this for any labelling, we will say the structure *B* is undetectable under the null model.

Working with the configuration null model, we derive general conditions first, then apply them to specific structures in the relevant sections. We first write the *Q*_*aa*_ and *Q*_*ab*_ matrix elements in terms of *S*_*aa*_, *S*_*ab*_ and *S*_*bb*_. The degree sum can be written as follows,

$$T_{a}=\sum_{i\in c,j}A_{ij}=\sum_{i\in a,j\in a}A_{ij}+\sum_{i\in a,j\in b}A_{ij}+\sum_{i\in a,j\notin a,b}A_{ij}=S_{aa}+S_{ab}+S_{a*}$$

where *S*_a*_ is the number of links from group *a* to all other groups apart from *a* and *b*. We also write

$$2E=S_{aa}+S_{bb}+2\left(S_{ab}+S_{a*}+S_{b*}\right)+S_{**}$$

Where S_**_ is twice the number of links in and between all groups apart from *a* and *b*. This gives

$$\begin{array}{c} Q_{ab}=S_{ab}-\frac{\left(S_{aa}+S_{ab}+S_{a*}\right)\left(S_{bb}+S_{ab}+S_{b*}\right)}{S_{aa}+S_{bb}+2\left(S_{ab}+S_{a*}+S_{b*}\right)+S_{**}} \end{array}$$

Some simple algebra the shows that the conditions for the matrix elements to be greater than zero can be written as

$$\begin{array}{c} Q_{aa}>0\equiv S_{aa}S_{**}>{\left(S_{ab}+S_{a*}\right)}^{2}-S_{aa}\left(S_{bb}+2S_{b*}\right) \end{array}$$ (9)

$$\begin{array}{c} Q_{ab}>0\equiv S_{ab}S_{**}>\left(S_{aa}+S_{a*}\right)\left(S_{bb}+S_{b*}\right)-S_{ab}\left(S_{ab}+S_{a*}+S_{b*}\right) \end{array}$$ (10)

Consider the ideal case where *a* and *b* can be connected to each other but share no links with the rest of the network. In this case *S*_a*_ = *S*_b*_ = 0 and the above inequalities simplify to

$$\begin{array}{c} Q_{aa}>0\equiv S_{aa}S_{**}>S_{ab}^{2}-S_{aa}S_{bb} \end{array}$$ (11)

$$\begin{array}{c} Q_{ab}>0\equiv S_{ab}S_{**}>S_{aa}S_{bb}-S_{ab}^{2} \end{array}$$ (12)

### 4.1 Core-periphery constraints

Consider a block matrix which has CP structure between groups labelled *c* and *p*, together with some other blocks that allow the KM rules to be satisfied. The block matrix to detect this looks like

$$B=(\begin{array}{ccccc} \vdots & \vdots & \vdots & \vdots & \vdots \\ \ldots & 1 & \ldots & 1\ldots \\ \vdots & \vdots & \vdots & \vdots & \vdots \\ \ldots & 1 & \ldots & -1\ldots \\ \vdots & \vdots & \vdots & \vdots & \vdots \end{array})$$

The groups *c* and *p* make a positive contribution to *Q*(*B*) if *Q*_*cc*_, *Q*_*cp*_ > 0 and *Q*_*pp*_ < 0. In the ideal case there are no internal links in the *p* group, so *S*_*pp*_ = 0, and there are no links between *c*, *p* and the rest of the network, any other labelling would make it more difficult to satisfy the sign conditions. In this case *Q*_*pp*_ < 0 and *Q*_*cp*_ > 0 always. However we only have *Q*_*cc*_ > 0 if

$$\begin{array}{c} S_{cc}S_{**}>S_{cp}^{2} \end{array}$$ (13)

otherwise *Q*_*cc*_ < 0. If *Q*_*cc*_ < 0 then we have a deficit of edges in the core compared to what would be expected under the null. In this case a block matrix with *B*_*cc*_ = −1 would give a larger value of *Q*(*B*), i.e. a bipartite (disassortative) relationship between *c* and *p* is detectable but a CP one is not. To summarize Eq 13, a core-periphery is detectable under the configuration model when

- The number of edges in the core, *S*_*cc*_, is large.
- The number of edges between core and periphery, *S*_*cp*_, is small.
- The core-periphery system is part of a larger network, S_**_ ≫ 0.

If these conditions are not met, there is no way to label the nodes of a network such that a sub-block is core-periphery under the configuration model. In the particular case discussed of a CP+1 community network, if that one community is reduced to a single node S_**_ = 0 and it is impossible to put any nodes into the periphery and still have a detectable structure.

### 4.2 Community-hierarchy constraints

The directed case can be analysed similarly and the equivalent of Eqs 11 and 12 for directed networks is

$$\begin{array}{c} Q_{aa}>0\equiv S_{aa}S_{**}>S_{ab}S_{ba}-S_{aa}S_{bb} \end{array}$$ (14)

$$\begin{array}{c} Q_{ab}>0\equiv S_{ab}S_{**}>S_{aa}S_{bb}-S_{ab}S_{ba} \end{array}$$ (15)

where it should be recalled that *S*_*ab*_ ≠ *S*_*ba*_ in general. CP structure is subject to the same constraints as in the undirected case, we need

$$\begin{array}{c} S_{cc}S_{**}>S_{cp}S_{pc} \end{array}$$ (16)

The community-hierarchy (CH) structure is the other interesting case. The corresponding block matrix is

$$B=(\begin{array}{ccccc} \vdots & \vdots & \vdots & \vdots & \vdots \\ \ldots & 1 & \ldots & 1\ldots \\ \vdots & \vdots & \vdots & \vdots & \vdots \\ \ldots & -1 & \ldots & 1\ldots \\ \vdots & \vdots & \vdots & \vdots & \vdots \end{array})$$

Consider groups *a* and *b* which have intra-community links and directed links from *a* to *b* only (so *S*_*ba*_ = 0) and are otherwise disconnected from the rest of the network.

Using the inequalities above we can show *Q*_*aa*_ > 0, *Q*_*bb*_ > 0 and *Q*_*ba*_ < 0 always. However *Q*_*ab*_ > 0 only if

$$\begin{array}{c} S_{ab}S_{**}>S_{aa}S_{bb} \end{array}$$ (17)

otherwise *Q*_*ab*_ < 0 and a simple two community structure (where *B*_*ab*_ = −1) is detectable while the community hierarchy is not. A community hierarchy can be detected if

- The number of edges between communities, *S*_*ab*_, is large.
- The number of edges in each community, *S*_*aa*_, *S*_*bb*_ is small.
- The community-hierarchy is part of a larger network, S_**_ ≫ 0.

### 4.3 Core-periphery example

To see what these limits look like in practice, consider an undirected network with three groups, a core *c*, a periphery *p* and a disconnected community *m*. Let there be an equal number, *N*, of nodes in each group. Construct a network using the Stochastic Block Model: every possible edge within and between groups is added with the corresponding probability from the matrix

$$\omega =(\begin{array}{ccc} p_{c} & p_{p} & 0\\ p_{p} & 0 & 0\\ 0 & 0 & p_{m} \end{array}).$$

These networks will be evaluated with two block patterns

$$Q_{CP}\equiv Q((\begin{array}{ccc} 1 & 1 & -1\\ 1 & -1 & -1\\ -1 & -1 & 1 \end{array}))$$

and

$$Q_{Bipartite}\equiv Q((\begin{array}{ccc} -1 & 1 & -1\\ 1 & -1 & -1\\ -1 & -1 & 1 \end{array}))$$

If the CP structure is detectable then *Q*_*CP*_ > *Q*_*Bipartite*_, where the nodes are labelled in the obvious way.

**Fig 3:**
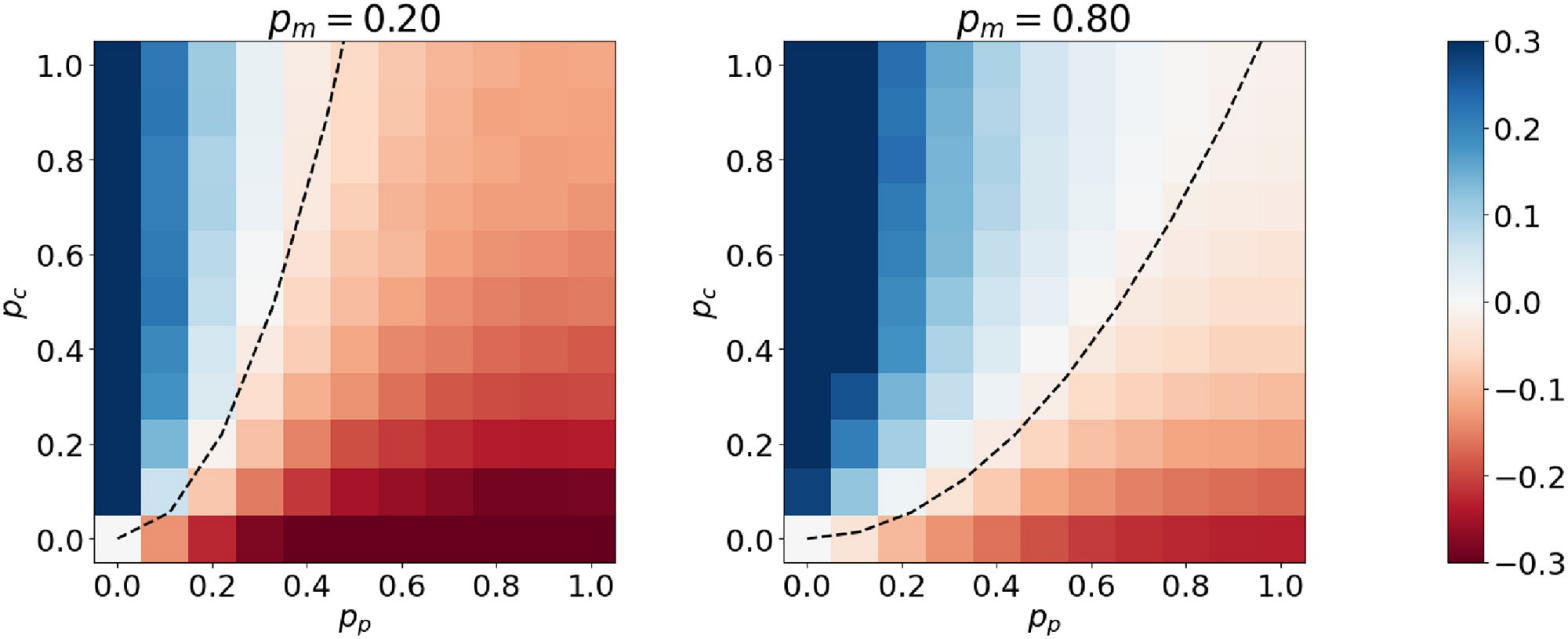
Color is *Q*_*CP*_ − *Q*_*Bipartite*_ for fixed *p*_*m*_ and variable *p*_*c*_, *p*_*p*_. Blue means CP can be detected under the configuration model, red means a bipartite structure is always preferred. The dashed line shows where *Q*_*CP*_ = *Q*_*Bipartite*_.

Fig 3 shows *Q*_*CP*_ − *Q*_*Bipartite*_ for fixed *p*_*m*_ as *p*_*c*_ and *p*_*p*_ varies. *N* = 30 and each pair of values is averaged over 20 random networks. *p*_*m*_ controls the size of the rest of the network and larger values give a larger region where CP structure can be detected. The dashed line shows where *Q*_*CP*_ = *Q*_*Bipartite*_, corresponding to the curve $p_{c}=\frac{p_{p}^{2}}{p_{m}}$. Pairs such as (*p*_*p*_, *p*_*c*_) = (0.4, 0.4) are notable as pairs of values of *p*_*c*_ and *p*_*p*_ where CP structure is detectable when the rest of the network is large (*p*_*m*_ = 0.8) and undetectable when it is small (*p*_*m*_ = 0.2).

Again note, this is not a result about maximising *Q*(*B*) to get the ‘right’ labels. This is a demonstration that even in the ideal case where edges are added according to the chosen pattern and the right labels are given we still can’t produce the desired pattern of excesses and deficits. For $p_{c}<\frac{p_{p}^{2}}{p_{m}}$ we could therefore say that the degree sequence alone explains the core-periphery structure.

### 4.4 Connection to the resolution limit

The well-known resolution limit of modularity [44] is a special case of these constraint equations. Consider two groups *a*, *b* which are part of a larger network. It is better, in terms of increasing *Q*(*B*), to merge *a* and *b* if *Q*_*ab*_ > 0. Repeating Eq 9 for convenience,

$$\begin{array}{c} Q_{ab}>0\equiv S_{ab}>\frac{\left(S_{aa}+S_{ab}+S_{a*}\right)\left(S_{bb}+S_{ab}+S_{b*}\right)}{2E} \end{array}$$

where the * index denotes all the nodes in the network other than the nodes in *a*, *b*. If there are no links between *a* and *b*, then *S*_*ab*_ = 0 and, provided there are some internal links in *a* or *b*, we can never have *Q*_*ab*_ > 0 and so the communities should be kept separate.

The resolution limit in [44] arises by considering a network with equal numbers of edges in *a* and *b*, one link between communities *a* and *b*, and one link connecting each of *a* and *b* to the rest of the network giving *S*_*ab*_ = *S*_a*_ = *S*_b*_ = 1 and *S*_*aa*_ = *S*_*bb*_ = 2*l*. Substituting into Eq 4.4 gives *Q*_*ab*_ < 0 if

$$l<\sqrt{E}-1$$

which is the result found in [44] and derived in a similar way to the above in [50]. As pointed out by [50], in the configuration model, when the network is large enough, the expectation for a single edge can be less than 1. The resolution limit is therefore modularity is doing what it is supposed to do, identifying that the single edge between communities is unusual under the null model. Eq 10 gives the most general form of the resolution limit. Disconnecting *a*, *b* from the rest of the network, which is the best case, two communities will be kept separate if

$$\begin{array}{c} S_{ab}S_{**}<S_{aa}S_{bb}-S_{ab}^{2} \end{array}$$ (18)

The threshold value of *S*_*ab*_ depends not only what is happening in the groups *a* and *b*, but also on what is happening in the rest of the network S_**_. Seen in this light, all of the results presented above are a type of resolution limit, but for core-periphery, community-hierarchy and other structures. Like the resolution limit for communities, they arise because the expected edge probabilities in large networks can become quite small in the configuration model [50]. The resolution limit suggests that, for small communities in large networks, even a single edge between ‘obvious’ communities can be unexpected. The results of the previous section similarly imply that ‘obvious’ CP and CH structures can be expected as a consequence of the degree sequence.

## 5 Nestedness

There are meso-scale structures which do not arise from 2 × 2 block patterns. Nestedness is one example, this is a network property often considered in the analysis of ecological networks [51] but with many other applications [45]. A nested network has a hierarchy of degrees such that the neighbors of a low degree node are a subset of the neighbours of any higher degree node [52]. A perfectly nested unipartite network admits an ordering of the nodes that brings the adjacency matrix into a triangular form. For example, the matrix

$$A=(\begin{array}{cccccccc} 0 & 1 & 1 & 1 & 1 & 1 & 1 & 1\\ 1 & 0 & 1 & 1 & 1 & 1 & 1 & 0\\ 1 & 1 & 0 & 1 & 1 & 1 & 0 & 0\\ 1 & 1 & 1 & 0 & 0 & 0 & 0 & 0\\ 1 & 1 & 1 & 0 & 0 & 0 & 0 & 0\\ 1 & 1 & 1 & 0 & 0 & 0 & 0 & 0\\ 1 & 1 & 0 & 0 & 0 & 0 & 0 & 0\\ 1 & 0 & 0 & 0 & 0 & 0 & 0 & 0 \end{array})$$

is perfectly nested. As noted in [53], this concept is very close to the idea of a core-periphery structure. Indeed in the ecological context [51], states, ‘To be nested, a network must consist of a core group of generalists all interacting with each other, and with extreme specialists interacting only with generalist species’, very similar to the definition of a core-periphery network.

By partitioning the nodes into two blocks, *c*, *p*, where nodes in *c* are highly connected (the bold upper left block) and rest of the nodes are in *p*, a nested network has the same block structure as a 2 × 2 core-periphery network. In practice we would not require perfectly nested structures, only that there is an excess or deficit of links compared the the expectation under the null model. The KM rules imply that, under the configuration model, such nested matrices cannot be constructed. Adding other structure to the network to avoid the KM rule leads to the CP constraint, Eq 13.

Nestedness is often studied in bipartite networks. For example, interactions between animals and plants, where no animal-animal or plant-plant interactions are considered. In this case the adjacency matrix has the form

$$A=(\begin{array}{cc} 0 & \tilde{A}\\ {\tilde{A}}^{T} & 0 \end{array})$$

Where $\tilde{A}$ is the *N* × *M* bi-adjacency matrix. In this context, a nested structure is one where $\tilde{A}$ has the distinct triangular form for some ordering of the nodes. Similarly to the above, we can split the bi-adjacency matrix into a core and periphery. A 4 × 4 block structure like

$$B=(\begin{array}{cccc} -1 & -1 & 1 & 1\\ -1 & -1 & 1 & -1\\ 1 & 1 & -1 & -1\\ 1 & -1 & -1 & -1 \end{array})$$

will have a positive *Q*(*B*) if the nodes can be labeled so that the adjacency matrix is bipartite and each part has a core that interacts with the other core and periphery and a periphery that only interacts with the other core. As this is a 4 × 4 block with a mixture of ±1 in each row, the KM rules are satisfied. Denote the left part by *a* and the right by *b*. *a* and *b* are then each split into core *c* and periphery *p* giving 4 groups: *ca*, *pa*, *cb*, *pb*. For this kind of structure, which would imply nestedness, to be detectable under the configuration model requires that *Q*_*ca*,*cb*_, *Q*_*ca*,*pb*_, *Q*_*cb*,*pa*_ > 0 and the rest are less than 0.

Assuming the best case of perfect bipartite structure and perfect CP structure between parts gives *Q*_*pa*,*pb*_ < 0, *Q*_*ca*,*pb*_ < 0 and *Q*_*cb*,*pa*_ < 0. The term *Q*_*ca*,*cb*_ is the interesting one. Using * = {*pa*, *pb*} gives S_**_ = 0, *S*_*ca*,*_ = *S*_*ca*,*pb*_ and *S*_*cb*,*_ = *S*_*cb*,*pa*_. Substituting into Eq 9 gives

$$Q_{ca,cb}>0\equiv S_{ca,cb}\left(S_{ca,cb}+S_{ca,pb}+S_{cb,pa}\right)>S_{ca,pb}S_{cb,pa}$$ (19)

This condition is somewhat more complicated that the CP and CH conditions, but broadly requires

- The number of interactions between the cores of *a* and *b*, *S*_*ca*,*cb*_, is large.
- The number of interactions between cores and peripheries, *S*_*ca*,*pb*_, *S*_*cb*,*pa*_ is small.

These results clarify the somewhat elusive nature of nestedness as highlighted in e.g. [54–57]. The configuration model is quite constraining. While an observed network may be sorted so that the adjacency matrix shows some structure, a random network with the same degree distribution could also be sorted to show similar structure. Under a less restrictive null model, like the ER model, the KM rules do not hold and nestedness is detectable, as found in e.g. [54]. However weakening the null risks confusing degree correlations for other structures [55], in particular dissassortative/bipartite ones. Work like [40], which situates nested structures inside larger networks is more promising for evading the KM rules and detecting nestedness under the configuration model.

**Fig 4:**
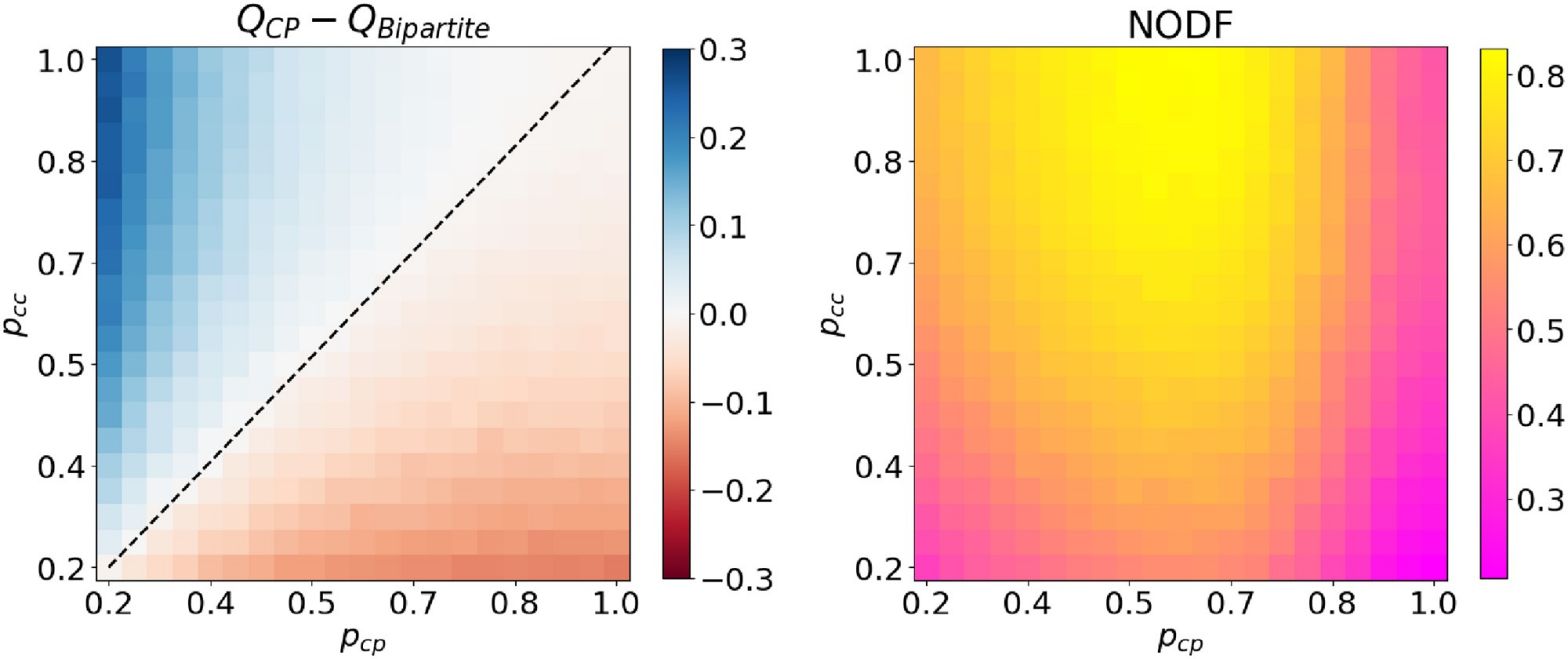
Left: *Q*_*CP*_ − *Q*_*Bipartite*_ networks constructed as described in the text. Blue means CP can be detected under the configuration model, red means a bipartite structure is always preferred. The dotted line shows where *Q*_*CP*_ = *Q*_*Bipartite*_. Right: NODF metric for the same networks. High values (near 1) imply a strongly nested network. The average of 20 random networks used for every *p*_*cp*_, *p*_*cc*_ pair.

Fig 4 shows what happens for networks constructed using the SBM where the edge probabilities are given by

$$\left(\begin{array}{cccc} 0 & 0 & p_{cc} & p_{cp}\\ 0 & 0 & p_{cp} & 0\\ p_{cc} & p_{cp} & 0 & 0\\ p_{cp} & 0 & 0 & 0 \end{array}\right)$$

placing *N*_*c*_ = 10 nodes in the core and *N*_*p*_ = 25 in the periphery. We evaluate block modularity against the patterns

$$Q_{CP}\equiv Q((\begin{array}{cccc} -1 & -1 & 1 & 1\\ -1 & -1 & 1 & -1\\ 1 & 1 & -1 & -1\\ 1 & -1 & -1 & -1 \end{array}))$$

and

$$Q_{Bipartite}\equiv Q((\begin{array}{cccc} -1 & -1 & -1 & 1\\ -1 & -1 & 1 & -1\\ -1 & 1 & -1 & -1\\ 1 & -1 & -1 & -1 \end{array}))$$

and evaluate nestedness using the popular NODF metric [58]. The key point is that there are cases where a Bipartite structure is preferred to a CP one but the NODF value is still quite high, for example, the point (*p*_*cp*_, *p*_*cc*_) = (0.7, 0.5). This would suggest that even though the network is apparently strongly nested, this is not surprising under the configuration null.

## 6 Relation of block modularity to the Stochastic Block model

The resolution limit is often partially evaded using the scaled configuration model [47]

$$P_{ij}=\gamma \frac{k_{i}k_{j}}{2E}$$

as a null model. It is shown in S1 File that the constraints, Eq 10, are similarly weakened with this null model. [43] (see also [59]) shows that maximising *Q*_*Newman*_ with this null model is equivalent to the method of maximum likelihood applied to a particular class of degree corrected Stochastic Block Models (dc-SBMs) [46] called the planted partition model *if γ is chosen correctly*. It turns out that block modularity with the scaled configuration model is similarly related to the dc-SBM. In fact, with a diffrent choice of null, block modularity can be made to be identical to the dc-SBM model likelihood in general.

In the dc-SBM, the edge probability is

$$P_{ij}=\frac{k_{i}k_{j}}{2E}\omega_{c(i),c(j)}$$

where *ω* is a *K* × *K* matrix controlling the expected number of edges in and between each group. In [43] it is shown that maximising the log-likelihood for the undirected dc-SBM corresponds to maximisation of the function

$$\begin{array}{c} \text{log}\;P\left(G|\omega ,c\right)=\frac{1}{2}\sum_{ij}(A_{ij}\;\text{log}\left(\omega_{c(i),c(j)}\right)-\frac{k_{i}k_{j}}{2E}\omega_{c(i),c(j)}) \end{array}$$ (20)

Where *P*(*G*|*ω*, *c*) is the probability of the observed network *G* given *ω* and the partition *c*. The planted partition model is one where

$$\omega_{ab}=\left(\omega_{in}-\omega_{out}\right)\delta_{ab}+\omega_{out}$$

corresponding to a matrix with *ω*_*in*_ on the diagonal and *ω*_*out*_ elsewhere. Substituting this into Eq 20 [43], shows that maximising *Q*_*Newman*_ is equivalent to maximising 20, provided the scaled configuration model is used with a particular value of *γ*. We can generalise this using our block matrix *B*. Define

$$\begin{array}{c} \omega_{ab}=\left(\omega_{in}-\omega_{out}\right)B_{ab}+\omega_{out} \end{array}$$ (21)

using values for *B* in {1, 0} in this case. The value *ω*_*in*_ is associated with the excesses and *ω*_*out*_ with the deficits. Substituting into Eq 20 rearranging and dropping some additive constants which don’t affect the optimisation gives

$$\begin{array}{c} \text{log}\;P\left(G|\omega ,c\right)=\frac{1}{2}\;\text{log}\;\frac{\omega_{in}}{\omega_{out}}\sum_{ij}B_{c(i),c(j)}(A_{ij}-\frac{k_{i}k_{j}}{2E}\frac{\omega_{in}-\omega_{out}}{\text{log}\;\omega_{in}-\text{log}\;\omega_{out}}) \end{array}$$ (22)

Which is equivalent to block modularity with the scaled configuration model, up to the scaling factor $\text{log}\;\frac{\omega_{in}}{\omega_{out}}$, *provided*

$$\gamma =\frac{\omega_{in}-\omega_{out}}{\text{log}\;\omega_{in}-\text{log}\;\omega_{out}}.$$

The standard configuration model has *ω*_*in*_ = *ω*_*out*_ = 1 which, due to the overall factor $\text{log}\;\frac{\omega_{in}}{\omega_{out}}$, causes Eq 22 to vanish. The likelihood, Eq 20, is derived by computing the probability of the network *G* given *ω* and *g*. With *ω*_*ab*_ = 1 knowing the partition gives no extra information about the probability of an edge existing and log *P*(*G*|*ω*, *c*) is constant.

Having one free parameter, *γ*, gives the model enough flexibility to be identified with the planted partition model. An even more flexible null model allows maximising block modularity to be equivalent to likelihood maximisation of the dc-SBM. First we rewrite the log-likelihood, Eq 20, as a sum over blocks

$$\begin{array}{c} \text{log}\;P\left(G|\omega ,c\right)=\frac{1}{2}\sum_{ab}(S_{ab}\;\text{log}\;\left(\omega_{ab}\right)-\frac{T_{a}T_{b}}{2E}\omega_{ab}) \end{array}$$

Now, define a new null model, which we will call the block scaled configuration model, via

$$P_{ij}=\frac{k_{i}k_{j}}{2E}\gamma_{c(i),c(j)}$$

Substituting into block modularity gives

$$\begin{array}{c} Q\left(B\right)=\frac{1}{2E}\sum_{ab}B_{ab}(S_{ab}-\frac{T_{a}T_{b}}{2E}\gamma_{ab}) \end{array}$$ (23)

If

$$B_{ab}=\text{log}\;\omega_{ab}$$

and

$$\gamma_{ab}=\frac{\omega_{ab}}{\text{log}\;\omega_{ab}}$$

then optimising the likelihood and optimising the modularity are equivalent. Here *ω*_*ab*_ is the edge probability relative to the configuration model. For *ω*_*ab*_ < 1, *B*_*ab*_ < 0 and *γ*_*ab*_ < 0 meaning *Q*_*ab*_ < 0, so it always reduces the modularity/likelihood and optimising the partition will involve trying to place fewer edges between *a* and *b*. For *ω*_*ab*_ > 1 we have *B*_*ab*_ > 0 and *γ*_*ab*_ > 0, so the modularity/likelihood can be increased by having more edges between *a* and *b* than the null model. This is similar to how we counted excesses and deficits in the block modularity with the configuration null model, except the excesses and deficits are now weighted.

Despite the possibility of making modularity optimisation equivalent to the maximisation of a dc-SBM likelihood, it is probably better to think of them as conceptually different approaches. In the ‘inferential’ approach [60], the parameters of the dc-SBM (node labels and edge probabilities) which could have generated the observed data are determined by maximising the likelihood. In the ‘descriptive’ approach taken here we probe for some fixed structure *B* by comparing the observed network to the expectation under some null hypothesis. To draw analogies with non-network statistics, this is like regression versus hypothesis testing. We compare these approaches on CP structure in the next section.

### 6.1 Inferred versus described CP structure

In the modularity framework there is no requirement that *B*_*ab*_ and *γ*_*ab*_ be related. If they are assumed to be related via *ω*_*ab*_ according to Eq 23, the values of *ω*_*ab*_ are not known and must be estimated. We could proceed as in [43] by choosing some starting values of *B* and *γ*; finding the partition that optimises the likelihood/modularity; estimate the dc-SBM parameters using

$$\begin{array}{c} \omega_{ab}=2E\frac{S_{ab}}{T_{a}T_{b}}; \end{array}$$ (24)

update *B* and *γ* and iterate the process until convergence. Preliminary experiments suggest this approach is quite sensitive to the starting values of *ω* e.g. if we start with nodes in assortative communities, it is very hard to transition to having the nodes in disassortative groups, even if this is optimal.

An approach that is sufficient for the small networks we will study here is a based on first fixing *K*, the number of groups. Estimating the correct number of groups is a difficult problem which we do not address, see e.g. [61]. Starting with random values of *ω*_*ab*_ = 1 ± 0.1 unif(−1, 1) and random labels *c*(*i*) ∈ {0, …, *K* − 1}, Eq 23 is maximised using a greedy label swapping heuristic. Visiting the nodes in random order, we compute the change in *Q* from swapping the label of *i* to any of the other allowed labels and choosing the swap which leads to the largest increase in *Q*. When computing the change caused by a label swap we consider the change in, *S*_*ab*_ and *T*_*a*_, as well as *ω*_*ab*_ using Eq 24, and consequently the change in *B*_*ab*_ and *γ*_*ab*_.

We will generate networks with known but undetectable patterns, then use this procedure (equivalent to fitting a SBM) to identify the node labels and edge probabilities *ω*. We will also infer the node labels and *ω* for random rewirings of the generated networks. Using a similar system to [19] we check 2 × 2 blocks of *ω* for structure using the following rules:

- First check if: *ω*_*aa*_ + 2*ω*_*ab*_ + *ω*_*bb*_ > 2*E*/2 i.e. the 2 × 2 block contains at least half of the network’s edges.
- Each pair of groups has the corresponding structure if one of the conditions below is satisfied:
  - Community: *ω*_*ab*_ < *α* min(*ω*_*aa*_, *ω*_*bb*_)
  - Bipartite: *αω*_*ab*_ > max(*ω*_*aa*_, *ω*_*bb*_)
  - CP: min(*ω*_*aa*_, *ω*_*bb*_) < *αω*_*ab*_ and min(*ω*_*aa*_, *ω*_*bb*_) < *α* max(*ω*_*aa*_, *ω*_*bb*_)

*α* ≤ 1 is a parameter where smaller values make the conditions more stringent.

**Fig 5:**
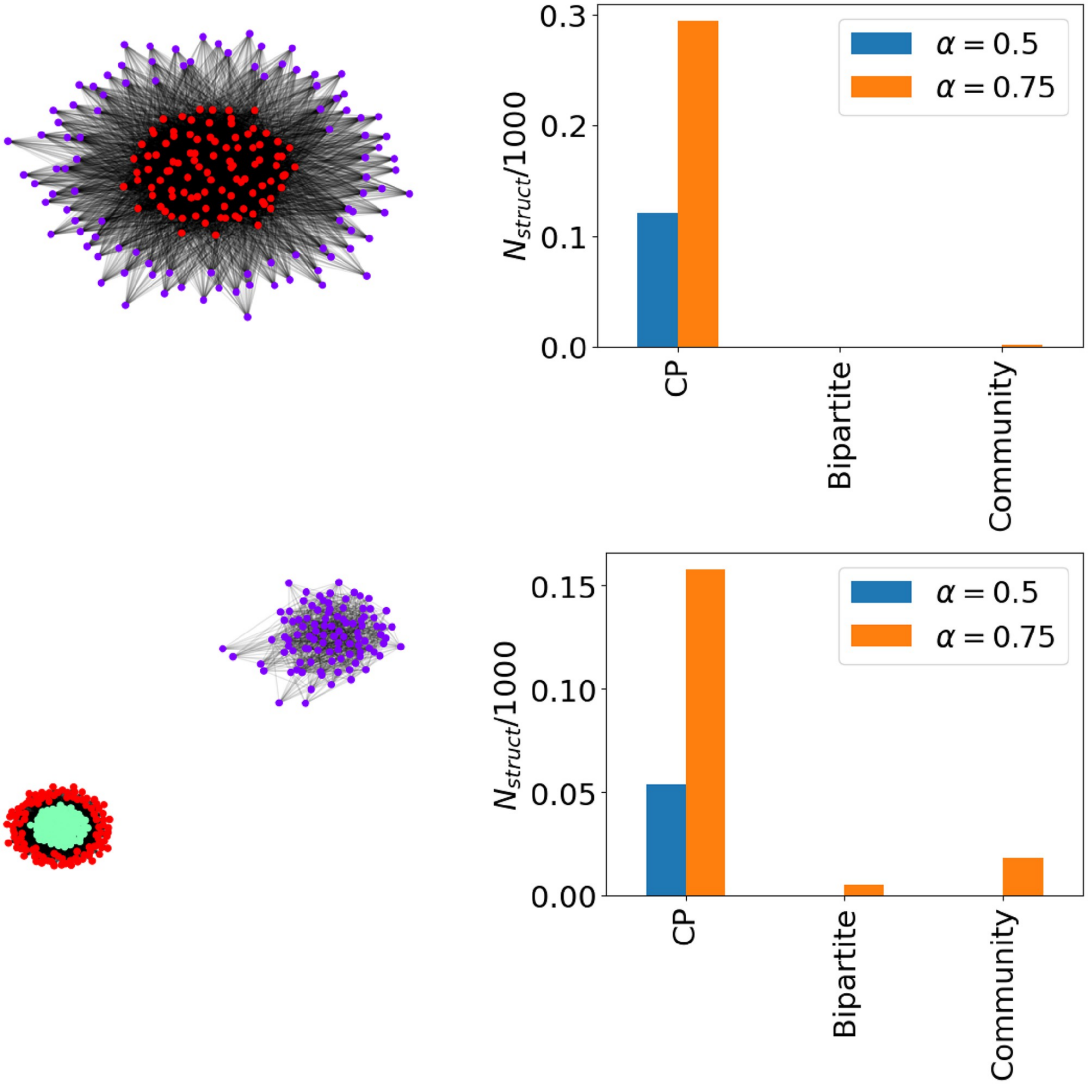
Left: SBM network, nodes coloured according to the partition inferred by maximising Eq 23. Right: Proportion of three structures found in 1000 samples from the configuration model of the left hand network. Top shows a CP network, bottom a CP+1 community network.

The top panel of Fig 5 shows this on a 2 × 2 CP structure and the bottom panel shows a CP+1 community structure generated with edge probabilities

$$\omega =(\begin{array}{cc} 0.8 & 0.4\\ 0.4 & 0 \end{array})$$

and

$$\omega =(\begin{array}{ccc} 0.6 & 0.5 & 0\\ 0.5 & 0 & 0\\ 0.0 & 0 & 0.2 \end{array})$$

respectively. The former is undetectable by the KM rules, the latter is undetectable by the constraint 13. The left panel shows that the optimisation of Eq 23 recovers the expected partition—we have fit a model to the data. When apply the same inference methods to networks with the same degree sequence as the networks on the left, the right panel shows that those random networks often have core-periphery structure. When we make the conditions more strict (lower *α*) we find fewer networks with any structure, though those that do are most often CP.

The effectiveness of inference of in the dc-SBM is often discussed in terms of efficiently compressing the network [62, 63] here we think of it as a model fitting process, where we find the partition and parameters that could be (and this case were) used to generate the network. In contrast, in the null model/modularity framework we are imagining that our network is one drawn at random from a fixed null model. We then ask about the chance of finding the same structure in a random network as seen in the observed one, finding that if any structure exists, it is most likely the planted one, implying the degree sequence alone is sufficient to create that structure.

## 7 Discussion

The results of Sections 3, 4 and 5 show that the configuration model is quite constraining in terms of the meso-scale structures that it allows. There are only two detectable 2 × 2 patterns, and there are strong constraints on the occurrence of CP and CH pairs as part of larger meso-scale structures. Nested networks are also quite restricted. Using a weaker null model, like the ER null (see S1 File)) relaxes these constraints but risks confusing real structure with the network’s degree sequence. An alternative approach, as discussed in Section 6, is based on supposing that some flexible model, usually the dc-SBM, generated the observed network and finding the most likely model parameters. The results of that section show that modularity maximisation is equivalent to model likelihood maximisation if the null model is also allowed to vary.

Paralleling vigorous debates in statistics over inferential Bayesian statistics versus frequentist methods [64, 65], the inferential approach has been strongly advocated [60, 66, 67] as superseding the ‘descriptive’ approach of modularity maximisation. This is motivated by the failure of modularity to produce intuitive partitions in some cases. As shown in this work, problems like the resolution limit are an example of a general type of constraint which arises when analysing network structure against a null model. Inequalities like Eq 10 and the resolution limit arise because the configuration model is quite stringent and intuitive structures, like core-periphery systems and nestedness can sometimes be explained by the degree sequence alone. If any random network with the same degree sequence as the observed network is likely to have similar ‘intuitive’ structure, the configuration model does not allow us to claim we found something. As in other cases [42, 56] the degrees of a network alone explain many apparently intuitive patterns and regularities.

It has been pointed out before that formalising the intuitive notion of ‘community’ or ‘structure’ is difficult [50] and a universal best definition is neither possible nor desirable [68]. For clustering non-network data, [69] suggests that clustering should not be treated as an abstract mathematical problem but evaluated in an application dependent way. Modularity is a particular formalization of the clustering problem: edges should be distributed in blocks in excess or deficit compared to a null model. The configuration null leads to constraints which rule out certain intuitive structures, in this particular formalization these structures are not meaningful. There are different solutions to this. One could accept it and stop looking for these structures, they are explained by the degree sequence, or one could abandon the formalization of excesses and deficits entirely, as advocated by [60], and use another one, usually fitting data to the SBM. As discussed by [32] however, there is ‘no free lunch’ [70], for example shows that inferential methods also fail to capture the intuitive structure in some cases.

There are also many modifications of the basic modularity definition which fix some or all of its issues, see [71–75] which correspond to alternative ways of counting excess and deficits, but are often somewhat *ad hoc*. There are also issues with how modularity is used in practice e.g. without properly accounting for statistical significance, however this can also be addressed [33]. We could also use a different null model, where the constraints are much weaker and CP and other structures are detectable, see the S1 File. The ER null is probably too weak and the scaled configuration model [47] has a arbitrary parameter to fix. As has been pointed out before [50] a configuration model that allows for some amount of variance in node degrees is likely to perform better than a model which fixes them exactly, but we leave this for future work.

The observation from [69]

People who work on end-use problems (“applications”) remain rather untouched by most of these papers. They continue to use their favorite algorithms, usually k-means and linkage algorithms.

seems to hold for network clustering, with “k-means and linkage” replaced by “Louvain and modularity maximisation”. [69] suggests the ultimate test of community or structure detection methods is their usefulness in practice. The aim of this work has been to extend the very popular modularity maximisation framework to non-assortative structures; understand the consequences of the commonly used and stringent configuration model on the detectability of such structures and show the connection between this framework and the popular alternative of SBM inference. I believe that extending, connecting and clarifying approaches to structure detection, giving practitioners a plurality of options for graph partitioning, is a more fruitful path to better results in applications than the search for a universal, optimal algorithm.

## Supporting information

S1 File Appendix. (ZIP)
