# Supplementary material for: Detectability constraints on meso-scale structure in complex networks
Source: PLoS One. 2025 Jan 22;20(1):e0317670. doi: 10.1371/journal.pone.0317670 (PMC11753644; doi:10.1371/journal.pone.0317670)
Supplement: S1 File — (ZIP) [file pone.0317670.s001.zip › appendix.pdf]

Detectability Constraints on Meso-Scale Structure in Complex Networks: Appendix

Rudy Arthur

University of Exeter, Department of Computer Science, Exeter, UK

R.Arthur@exeter.ac.uk

A Other Null Models

A.1 Erdős-Rényi Model

With the ER null model the global sum provides a hard constraint

$$\begin{aligned}\sum_{ab} Q_{ab} &= \sum_{ab} S_{ab} - \sum_{ab} pN_a N_b \\ &= 2E - pN^2 = 0\end{aligned} \tag{1}$$

where N_a is the number of nodes in group a. This means under the ER null model we can have an excess (or deficit) between one group and all the others (including itself), but we can't have an excess or deficit between every group with every other one. This rules out the all white or all black block patterns. The row sums are

$$\sum_b Q_{ab} = \sum_b S_{ab} - N_a p \sum_b N_b = T_a - 2E\frac{N_a}{N} \tag{2}$$

So if there is a group with all excesses, this is an upper limit on the contribution of that group to the modularity, and similarly for deficits, the negative of the above gives a lower bound. The equivalent of Eq 10 is

$$Q_{ab} > 0 \equiv \frac{S_{ab}}{2E} > \frac{N_a N_b}{N^2} \tag{3}$$

i.e. the fraction of edges observed between or within groups a and b should be greater than the fraction of edges which could possibly exist.

A.2 Scaled Configuration Model

The row sums for the scaled configuration model are

$$\sum_b Q_{ab} = T_a(1-\gamma) \tag{4}$$

and the total sum is

$$\sum_{ab} Q_{ab} = 2E(1-\gamma) \tag{5}$$

The value of γ sets the upper and lower limits on all black or all white rows. For overcoming the resolution limit values of $\gamma > 1$ are usually proposed [1]. However for detecting CP structure, $\gamma < 1$ is preferable. The equivalent of Eqs 11 and 12 is

$$Q_{ab} > 0 \equiv S_{ab}S_{**} > \gamma(S_{aa}S_{bb} - S_{ab}^2) + (\gamma - 1)S_{ab}(S_{aa} + 2S_{ab} + S_{bb}) \quad (6)$$

$$Q_{aa} > 0 \equiv S_{aa}S_{**} > \gamma(S_{ab}^2 - S_{aa}S_{bb}) + (\gamma - 1)S_{aa}(S_{aa} + 2S_{ab} + S_{bb}) \quad (7)$$

The scaled configuration model can be treated by substituting $\gamma \to \gamma_{ab}$ in the equations above. For CP structure requiring $Q_{cc} > 0$ implies

$$Q_{cc} > 0 \equiv S_{cc}S_{**} > \gamma S_{cp}^2 + (\gamma - 1)S_{cc}(S_{cc} + 2S_{cp}) \quad (8)$$

so $\gamma < 1$ makes CP structure easier to achieve under this model. Similar results to the ones found in Sections 4.4 and 5 can be derived using the above equations for this null.

References

1. Andrea Lancichinetti and Santo Fortunato. Limits of modularity maximization in community detection. *Physical review E*, 84(6):066122, 2011.
